# Transcriptome profile of delayed mortality in patients with invasive group A Streptococcal disease

**DOI:** 10.1371/journal.pone.0356286

**Published:** 2026-08-18

**Authors:** Ville Kailankangas, Shintaro Katayama, Kirsi Gröndahl-Yli-Hannuksela, Johanna Vilhonen, Mari Hannele Tervaniemi, Kaisu Rantakokko-Jalava, Tapio Seiskari, Emilia Lönnqvist, Juha Kere, Jarmo Oksi, Jaana Syrjänen, Jaana Vuopio

**Affiliations:** 1 Faculty of Medicine and Health Technology, Tampere University, Tampere, Finland; 2 Department of Internal Medicine, Tampere University Hospital, Tampere, Finland; 3 Folkhälsan Research Center, Helsinki, Finland; 4 Stem Cells and Metabolism Research Program, University of Helsinki, Helsinki, Finland; 5 Department of Medicine Huddinge, Karolinska Institutet, Huddinge, Sweden; 6 Institute of Biomedicine, University of Turku, Turku, Finland; 7 Department of Infectious Diseases, Turku University Hospital, Turku, Finland; 8 Department of Clinical Microbiology, Laboratory Division, Turku University Hospital, Turku, Finland; 9 Department of Clinical Microbiology, Fimlab Laboratories, Tampere, Finland; 10 Faculty of Medicine, University of Turku, Turku, Finland; 11 Finnish Institute for Health and Welfare (THL), Helsinki, Finland; Centers for Disease Control and Prevention, UNITED STATES OF AMERICA

## Abstract

**Background:**

Our aim was to study the pathophysiological mechanisms of early (<7 days) and delayed sepsis mortality by transcriptome profiling of cases with invasive group A Streptococcal (iGAS) infections and possibly find predictors for delayed mortality.

**Methods:**

We recruited cases with iGAS from June 2018 to June 2020. Whole blood samples were collected at the early timepoint (within two days after admission, timepoint A) and later timepoint (a week later, timepoint B) to perform transcriptome analysis. A comparison of gene expression against disease course was done using weighted genome correlation network analysis.

**Results:**

We recruited 45 patients. Eight patients died within 90 days, four of whom within the first week. After RNA quality control 34 and 31 subjects at timepoints A and B remained, respectively. The gene expression profiles associated with a severe disease course differed markedly between timepoints A and B. High expression of necroptosis factors and low expression of HLA genes at timepoint B was associated with death.

**Conclusions:**

The seemingly different gene expression profile over time among patients with a severe iGAS disease may suggest distinct pathophysiological mechanisms for early and delayed sepsis mortality. However, as our sample size was small, these findings must be considered with caution and should not be interpreted as predictive.

## Introduction

*Streptococcus pyogenes*, or Group A *Streptococcus* (GAS), is an important human pathogen, that can cause severe and life threatening invasive (iGAS) infections [1]. Host genetics are assumed to affect the development of severe invasive disease [2].

Transcriptome analysis of peripheral blood has been demonstrated to be of use in describing the host immune response in septic patients [3]. Weighted gene correlation network analysis (WGCNA) is a bioinformatics algorithm that can augment transcriptome analysis by grouping genes involved in the same cellular processes and with similar functions according to co-regulation. New functions of such groups, or modules, of genes can be inferred by relation to one another and external sample traits. Genes in a module correlated to a trait are also biomarker candidates [4].

Most sepsis deaths occur in the first days, with up to a half estimated to occur in the first weeks [5]. The timing of mortality for iGAS infections is similar [6]. However, a significant number of deaths occur even if a patient survives the critical first week. It has been postulated, that there may be distinct pathophysiological mechanisms behind sepsis deaths during the first week and deaths that happen 8–90 days after the onset of the illness [7]. Survivors of acute sepsis may develop a chronic critical illness, which results in delayed mortality and adverse long-term outcomes [8].

Higher Charlson comorbidity index scores and a higher sequential organ failure assessment score at 72 hours have been shown to predict chronic critical illness and delayed mortality [9], but this data is for surgical intensive care unit patients. There is also evidence that the transcriptome profiles of acute sepsis and chronic critical illness are markedly different [8].

In this study we set out to compare gene expression profiles of iGAS patients in the first days of their illness and during the second week of their illness. We hypothesized that the gene expression profile would differ between the cases who died in the first week of the illness and the ones that died at a later date, and that they could perhaps be used to augment prediction models for delayed mortality.

## Materials and methods

The details of the study design have been explained previously [10]. In brief, we prospectively recruited cases with an invasive GAS infection at two Finnish hospitals, Tampere University Hospital and Turku University Hospital, between 30^th^ of June 2018 and 2^nd^ of June 2020. A case had to be culture positive for GAS from blood, CSF, pleural fluid, peritoneal fluid, synovial fluid or deep tissue sample, i.e., a normally sterile site, and over 18 years of age.

For the purposes of this study, a whole blood sample for RNA isolation (PAXgene Blood RNA tube, BD) was taken of all cases at two timepoints: when first recruited, when the causative pathogen was confirmed to be GAS (on average two days after admission, timepoint A) and five to seven days later (timepoint B).

Case interviews and electronic patient records were used to obtain background data and laboratory test results with the cases’ informed consent. All data were pseudonymized with subject codes.

The methodology for the transcriptome analysis and RNA sequencing has also been described in our previous publication [11] focusing on transcriptomic models of the early stage in severe iGAS. Briefly, the corrected expression levels were normalized by spike-in controls and varied endogenous protein coding genes were selected at each timepoint by comparing to the technical variations as estimated by the spike-ins (p < 0.05, adjusted by Benjamini-Hochberg procedure) [11]. Then WGCNA was used to classify genes that had significantly altered expression in at least one timepoint, and to estimate associations between expression profiles and selected clinical traits. The soft threshold used for the WGCNA was 16, the p-values were unadjusted. Gene ontology enrichment was done using Enrichr [12] and gene ontology p-values were adjusted using the Benjamini-Hochberg procedure. In parallel, HLA typing was done by arcasHLA [13] and the IMGT/HLA database version 3.34.0 [14]. Associations of the observed HLA alleles to clinical traits were evaluated by Boruta [15]. The selected comparators were need for intensive care, death, and severe disease (a composite of the prior two). Factors that could confound the analysis, such as age, gender, and underlying conditions as defined by Charlson comorbidity index were also compared [16].

Unpaired, nonparametric Mann-Whitney U test (IBM SPSS Statistics) was used to compare differences between the cases who died after timepoint B and those who survived.

The study protocol was approved by the Regional Ethics Committee of the Expert Responsibility Area of Tampere University Hospital and local research permissions were obtained from both research hospitals accordingly (permission numbers R18062, T05/026/18). The study was registered at ClinicalTrials.gov as ID NCT03507101. This study was conducted in accordance with the Declaration of Helsinki. Written, informed consent was obtained from all study participants or from next of kin for sedated or intubated patients.

### Definitions

We defined severe disease as needing intensive care or ending in death. Early mortality was defined as occurring before timepoint B (on average 7 days after timepoint A), and delayed mortality as occurring after timepoint B but within 90 days. Charlson Comorbidity Index was used to classify the patients’ underlying characteristics, and further subdivided into four classes, 0 score is 0, 1–2 scores are 1, 3–4 scores are 2 and ≥ 5 is 3 [16].

## Results

A total of 45 patients were recruited. Timepoint A was an average of 4.5 days after the onset of fever (range −2–11 days, interquartile range four days), and two days after admission (range 1–10 days, interquartile range two days), while timepoint B was on average 7 days after timepoint A (range 7–9 days, interquartile range 0 days). A thorough outline of clinical characteristics and foci of infection has been published previously [10]. There were 27 (60%) male and 18 (40%) female cases respectively, with a mean and median age of 55 years. The most common Charlson class was class 1, with 42% (19/45) of cases, while class 0 had 20% (9/45), class 2 13% (6/45) and class 3 24% (11/45). A need for intensive care occurred in thirteen cases (29%), and overall mortality was 18%. Four patients died during the first week of hospitalization, three after timepoint B, but still during hospitalization and one over a month after the onset of the disease, but within 90 days of onset. The survival or non-survival of all cases was ascertained from their electronic patient records, which are linked to the Finnish Population Information System.

The cases who died after timepoint B had a higher C-reactive protein level on admission (274 mg/l, 95% CI 168–382 mg/l versus 180 mg/l, 95% CI 144–217 mg/l, p = 0.18) than those who survived, but the difference was not statistically significant. Nor was the difference in leukocyte count at admission (mean 13.0E9/l, 95% CI 11.2-14.8E9/l for the survivors and 23.5E9/l, 95% CI 9.3-37.6E9/l for the cases who died after timepoint B, p = 0.29) or C-reactive protein level at timepoint B (52 mg/l, 95% CI 35–68 mg/l for the survivors and 80 mg/l, 95% CI 0–169 mg/l for the cases who died after timepoint B, p = 0.38). The cases who died after timepoint B were older (mean 78 years vs 53 years, p = 0.014) and had a higher Charlson comorbidity score (mean 7.5 vs 2.4, p = 0.001) than the survivors. There was no difference in the delay from the onset of fever to timepoint A between survivors and non-survivors (p = 0.17).

Blood samples for RNA extraction were acquired from 41 cases at timepoint A and 35 at timepoint B. After disqualifying samples with assay failure, we had 34 and 31 subjects remaining at timepoints A and B, respectively. All the cases who died after timepoint B were included.

**Fig 1:**
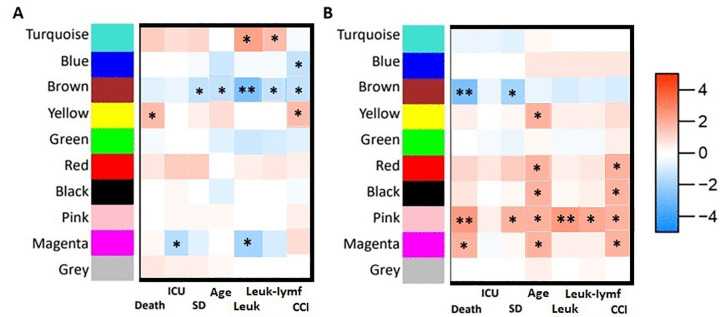
Associations of the modules with correlating gene expression levels, and the clinical traits at timepoints A (Fig 1A) and B (Fig 1B). All genes are reassigned by WGCNA according to co-regulation for each timepoint, so the composition of the modules is reset. The color gradient signifies values of -log10(P) * sig(r), where P is the unadjusted P-value of a correlation coefficient between expression levels of a module and clinical parameters, r is the correlation coefficient, and sig(r) is 1 if r > 0 (= positive correlation) and −1 if r < 0 (= negative correlation). The asterisks signify statistical significance, * = p < 0.05, ** = p < 0.005. ICU = intensive care unit, SD = severe disease (a composite of the death and ICU categories), Leuk = total leukocyte count, Leuk-lymf = total leukocyte count minus lymphocyte count, CCI = Charlson comorbidity index.

The genes with biological expression variation in the qualified samples at each timepoint were classified into ten modules by WGCNA according to the correlation. The modules were identified with a randomly selected color code. This was done for each timepoint separately, with the genes reassigned to modules in each timepoint according to co-regulation at that timepoint. The genes with the strongest association to severe disease differed between the timepoints. At timepoint A (Fig 1A), high expression of the genes in the yellow module was associated with death at any stage (p < 0.05), whereas low expression of the genes in the magenta and brown modules was associated similarly with need for intensive care and severe disease respectively (both p < 0.05), but not strongly associated with death alone. Low expression of the genes in the brown module was also associated inversely with leukocyte count and total leukocyte count minus lymphocyte count. No module had significant associations to the focus of infection.

At timepoint B, low expression of the brown module and high expression of the pink module had a strong correlation (p < 0.01) with delayed death (Fig 1B). Both were also associated (p < 0.05) with the composite category of severe disease, but neither with need for intensive care alone, although the pink module was also associated with the need for respiratory support. High expression of the pink module also correlated with advanced age and higher Charlson class, as well as higher total leukocyte count, and a higher total leukocyte count minus lymphocyte count.

**Fig 2:**
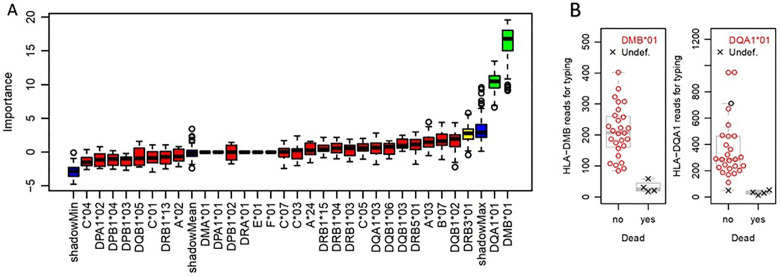
Association of expressed HLA-HMB and -DQA1 with death. (A) Importance scores of HLA types on the association with death. Shadows (blue) are references made by randomly shuffled data. Confirmed HLA types (green) had higher importance score than the shadows.(B) RNA-seq read counts on the confirmed HLA types and the association with death. Undef represents HLA typing failure, which may imply novel types or may be due to lower expression; therefore, for example, black dots in HLA-DQA1 represent other than DQA1*01.

The pink module contained genes relating to neutrophil regulation and necroptosis, whereas the brown module had many HLA genes (HLA-C/-DMA/-DMB/-DOB/-DPA1/-DPB1/-DQA1/-DRA/-DRB1/-DRB5) (Tables 1 and 2). Especially HLA-DMB*01 and HLA-DQA1*01 showed importance in association with death (Fig 2).

**Table 1:** Genes with the strongest association to death in the brown and pink modules at timepoint B.

Brown	AUC	95% CI	Pink	AUC	95% CI
SDAD1	1.00000	1.000-1.000	**SLPI**	1.00000	1.000-1.000
**HLA-DRB1**	0.99074	0.965-0.990	FOXP1	0.98148	0.937-0.981
BIN1	0.98148	0.937-0.981	H2AC13	0.96296	0.896-0.963
EVL	0.98148	0.937-0.981	ARG1	0.95370	0.879-0.954
LY86	0.98148	0.937-0.981	FLOT1	0.95370	0.878-0.954
AHNAK	0.98148	0.940-0.981	IL1R2	0.95370	0.871-0.954
DNPH1	0.98148	0.940-0.981	YAF2	0.94444	0.861-0.944
EXOSC5	0.98148	0.940-0.981	IRAK3	0.93519	0.820-0.935
HLA-DPA1	0.98148	0.940-0.981	ANXA3	0.92593	0.829-0.926
ORC5	0.98148	0.940-0.981	PTEN	0.92593	0.793-0.926

In the brown module the association is to low expression, and in the pink module to high expression. AUC = area under the receiver operating characteristic curve. The four cases that died after timepoint B constitute the sample.

**Table 2:** Top 10 gene ontology biological process terms for pink (first ten) and brown (lower ten) module genes at timepoint B.

Term	Overlap	Adjusted P-value	Odds Ratio
**neutrophil** degranulation (GO:0043312)	13/481	3.59E-07	11.7
**neutrophil** activation involved in immune response (GO:0002283)	13/485	3.59E-07	11.6
**neutrophil** mediated immunity (GO:0002446)	13/488	3.59E-07	11.5
response to lipid (GO:0033993)	6/114	1.82E-04	20.7
regulation of toll-like receptor signaling pathway (GO:0034121)	4/27	1.82E-04	62.9
response to molecule of bacterial origin (GO:0002237)	5/73	3.22E-04	27.0
positive regulation of DNA-binding transcription factor activity (GO:0051091)	7/246	8.82E-04	11.0
regulation of programmed **necrotic cell death** (GO:0062098)	3/20	0.00267556	62.7
regulation of **necroptotic process** (GO:0060544)	2/23	0.00367149	53.3
positive regulation of toll-like receptor signaling pathway (GO:0034123)	2/27	0.0054119	44.4
**Term**	**Overlap**	**Adjusted P-value**	**Odds Ratio**
ribosome biogenesis (GO:0042254)	41/192	1.44E-19	9.06585725
rRNA processing (GO:0006364)	38/173	9.89E-19	9.35743619
rRNA metabolic process (GO:0016072)	36/162	5.33E-18	9.46987952
translation (GO:0006412)	39/214	1.63E-16	7.40595156
ncRNA processing (GO:0034470)	37/201	7.86E-16	7.47585156
peptide biosynthetic process (GO:0043043)	33/162	1.99E-15	8.43397579
SRP-dependent cotranslational protein targeting to membrane (GO:0006614)	25/90	7.71E-15	12.5506757
cytoplasmic translation (GO:0002181)	25/93	1.60E-14	11.9951063
protein targeting to ER (GO:0045047)	26/103	1.70E-14	11.030303
cotranslational protein targeting to membrane (GO:0006613)	25/94	1.70E-14	11.8206522

Overlap means the overlap rate with member genes of the gene ontology term, the denominator means the number of total genes with which the biological term is associated, and the numerator is the number of genes in the module associated with the term. P-values adjusted with Benjamini-Hochberg.

The yellow module of timepoint A, that was associated with death, only had an overlap of one gene with the brown module of timepoint B, and no overlap with the pink module of timepoint B. The brown and magenta modules of timepoint A, that had an association with severe disease, but not with death, had 330 overlapping genes with the brown module of timepoint B, but only one with the pink module of timepoint B. Among the genes with the strongest association (table 1), there was no overlap. Overlap was tested with Venny [17].

## Discussion

Our understanding of the pathophysiology of sepsis and the inflammatory mechanisms involved have increased greatly in recent years with the advent of metagenomics, but much is still unclear. Up to almost half of sepsis deaths happen in the first week after onset [7]. In a study of patients with surgical sepsis, mortality was 4% within the first 14 days, and patients who survived this period either recovered or developed what was termed chronic critical illness. Mortality was 20% among chronic critical illness patients [9]. Another study of four patients with sepsis conducted 14–21 days after onset demonstrated unique transcriptomic patterns of circulating immune cells when compared to healthy controls [8]. Upregulation of many HLA genes was demonstrated in the cases with chronic critical illness.

Contrarily, in our study it was downregulation of many HLA genes that seemed to be associated with disease severity and death at timepoint B. This discrepancy can be explained by the small sample sizes in both studies, but can also be hypothesized to demonstrate a behavioral trend of HLA regulation over time, as the timepoints of the above study differed markedly from our timepoint B, with our timepoint B being 7–14 days earlier than the transcriptome studies conducted in the above study [8].

Interestingly, the HLA gene expression did not appear to be associated with advanced age, a known risk factor for chronic critical illness [9]. In our previous article [11], we showed downregulation of various genes of cytotoxic immunity to be associated with severe disease in the early stage. At timepoint A, no HLA types had a strong association with disease severity. None of the genes most strongly associated with disease severity at timepoint A had this same association at timepoint B, and when looking at only death, there was an overlap of only one gene.

The cases who survived until timepoint B then seem to have either reached a more homeostatic state of immunomodulation or they may have a persistent low expression of differing lymphocyte functions, including antigen presentation, which seems to be associated with a poor prognosis. The module with overexpression of neutrophil regulators and necroptosis regulators having an association with death and severe disease could be seen to support the theory of an ongoing dysregulated immune response as a factor of poor prognosis. In another study of patients with septic shock, low monocyte HLA-DR expression at 3–4 days after onset was associated with mortality [18], which aligns with this possibility. The finding that this module was also associated with higher total leukocyte count and a higher total leukocyte count minus lymphocyte count seems logical, as higher neutrophil counts would likely promote higher neutrophil regulation. This could also point to immune dysregulation. At timepoint A, the relationship seemed inverse, with low expression of neutrophil regulatory factors in the brown module correlating with severe disease.

The difference in expression profiles between timepoints A and B could point to differing mechanisms of mortality in the very first days of severe invasive infection and in the weeks that follow. Both perhaps represent a failure of immunomodulation. Similar inferences are suggested by Monneret et al in the study on septic shock patients [18]. No difference was seen in commonly used markers of inflammation between survivors and those who died after timepoint B in our study, but this is likely also due to our small study population.

Advanced age and a higher Charlson class were associated with overexpression of the module containing neutrophil and necroptosis regulatory factors, which was also associated with delayed death. This is logical, as older age and comorbidities are risk factors of chronic critical illness [10], and the cases that perished after timepoint B were significantly older than the survivors. It is possible, that the correlation with death is a byproduct of this association. It could also be a sign of dysfunctional immunomodulation relating to advanced age and chronic illness, and as such part of the mechanism of the risk associated with advanced age and comorbidities. This module was also associated with higher overall leukocyte count and higher total leukocyte count minus lymphocyte count. Accordingly, the preponderance of such gene expression can be due to the neutrophil cell line being overrepresented.

The main weakness of this study is the small study population. Ideally, the number of events per variable for a robust logistic regression analysis of gene expression would be 10 [19]. The number of cases who died after timepoint B was only four, which renders our findings only exploratory in nature, and as such the results presented here are merely suggestive and no causality or predictive factors can be reliably inferred. Our findings can thus be seen as a hypothesis needing validation in a larger cohort. Another limitation is the lack of cases with invasive disease caused by other pathogens, as our study protocol focused on GAS. Therefore, we cannot know if this gene expression behavior would present universally in severe infections. Furthermore, whole-blood RNA-seq does not distinguish per-cell expression from changes in the proportions of leukocyte populations, and as such the possibility that our findings merely reflect changes in relative cell population sizes cannot be ruled out.

## Conclusions

In our study, the gene expression profile as evidenced by transcriptome WGCNA appeared to behave differently over time among patients with a severe iGAS disease leading to death. This could be indicative of differing pathophysiological mechanisms for early and delayed sepsis mortality, but this is only an exploratory, suggestive finding, and a larger study cohort is needed for validation.
