# Supplementary material for: Transcriptome profile of delayed mortality in patients with invasive group A Streptococcal disease
Source: PLoS One. 2026 Aug 18;21(8):e0356286. doi: 10.1371/journal.pone.0356286 (PMC13484954; doi:10.1371/journal.pone.0356286)
Supplement: Supplementary file 1 [file pone.0356286.s001.docx]

Comments

- Only four patients experienced delayed death (after timepoint B), yet multiple gene modules, individual genes, and HLA types are evaluated in relation to this outcome. The authors themselves cite Peduzzi et al. and acknowledge that the events-per-variable ratio required for robust modeling is not met. In its current form, the risk of overfitting and chance associations is very high. Please substantially temper all causal and predictive claims regarding delayed mortality, explicitly frame findings as exploratory/hypothesis-generating, and avoid language implying that these modules “differentiate delayed death” with clinical reliability. The ROC/ AUC values of 0.95–1.00 for individual genes/modules with only four events are not credible as stable estimates. These require clear reporting of CI, emphasis on overfitting and optimism bias and removal of any suggestion that these are ready for clinical risk stratification.
- Patients who died after timepoint B were significantly older and had markedly higher Charlson comorbidity scores than survivors (mean age 78 vs 53 years, Charlson 7.5 vs 2.4). These factors are strong predictors of delayed mortality and may drive both the clinical outcome and aspects of the transcriptome. The current analysis appears largely unadjusted for these key confounders.
- The Abstract and Conclusions state that gene expression “was better at differentiating delayed death from survival than C-reactive protein level.” Yet there is no formal comparison of discrimination (e.g., AUCs with CIs for module vs CRP, or net reclassification) and CRP differences are described only descriptively and non-significant.
- The Discussion notes that prior work in chronic critical illness found upregulation of many HLA genes, whereas this study finds downregulation of multiple HLA genes at timepoint B associated with severity and death. This apparent discrepancy deserves deeper exploration.
- A more nuanced, mechanistic discussion would improve the translational value and help avoid overinterpretation.
- The authors acknowledge that whole-blood RNA-seq cannot distinguish per-cell expression from changes in leukocyte populations. This is particularly relevant because modules linked to delayed death at timepoint B relate to neutrophil regulation, necroptosis, and HLA expression and are associated with leukocyte count and right shift.
- Explore whether simple measures such as neutrophil and lymphocyte counts (or NLR) correlate with the key modules and whether modules remain associated with outcome after adjusting for these counts in a univariable or limited multivariable framework.
- Emphasize in the Discussion that some transcriptomic signals may primarily reflect shifts in cell populations rather than intrinsic reprogramming.
- Provide thresholds for inclusion of genes (filtering criteria for low expression/variance) and the soft-thresholding power used in WGCNA, including rationale.
- Clarify module preservation/overlap between timepoints A and B; the text states that genes were reassigned at each timepoint, but some quantitative measure of overlap would help interpret the “markedly different” profiles.
- Multiple testing: clarify how multiple comparisons were controlled across modules, traits, and genes (beyond the -log10(P)*sign(r) metric). Were adjusted p-values or FDRs calculated for module–trait associations and GO analyses?
- For the Mann–Whitney U tests comparing laboratory values between survivors and delayed deaths, provide exact p-values and 95% CIs where possible.
- Please clarify:
  - The distribution of time from symptom onset and from admission to sampling at timepoints A and B (medians and IQRs).
  - How deaths occurring “later in hospital” and “over a month after onset” are allocated into early vs delayed categories.
  - Whether any patients were lost to follow-up between discharge and 90 days, and how this was handled.
- For Table 1, report 95% CIs for the AUCs and sample sizes used.
- For Table 2, briefly indicate how GO enrichment p-values were adjusted (e.g., Benjamini–Hochberg).

**Recommendation**

In summary, the study raises an interesting and clinically relevant hypothesis about distinct transcriptomic patterns associated with delayed mortality in iGAS sepsis, but the very limited number of delayed death events and lack of robust adjustment for confounding restrict the strength of the conclusions. I would support publication in PLOS ONE after major revision that (1) reframes the work as exploratory, (2) clearly presents the limitations and potential biases, and (3) strengthens methodological transparency and interpretative restraint as outlined above.
