# Supplementary material for: Transcriptome profile of delayed mortality in patients with invasive group A Streptococcal disease
Source: PLoS One. 2026 Aug 18;21(8):e0356286. doi: 10.1371/journal.pone.0356286 (PMC13484954; doi:10.1371/journal.pone.0356286)
Supplement: Supplementary file 2 [file pone.0356286.s003.docx]

Dead editor

Below are our responses for the points raised by the PLOS ONE editors, as well as those by the reviewers. Our responses are written in cursive after each point.

1. Please ensure that your manuscript meets PLOS ONE's style requirements, including those for file naming. The PLOS ONE style templates can be found at
[https://journals.plos.org/plosone/s/file?id=wjVg/PLOSOne_formatting_sample_main_body.pdf](https://track.editorialmanager.com/CL0/https:%2F%2Fjournals.plos.org%2Fplosone%2Fs%2Ffile%3Fid=wjVg%2FPLOSOne_formatting_sample_main_body.pdf/1/010f019d6117a4df-f456d06a-45cc-4755-b303-5bc0431b0a28-000000/nffhZk9J-kD3Mx3yEnVXbUxCyHYXapfLSzmsWBNkfO8=257) and [https://journals.plos.org/plosone/s/file?id=ba62/PLOSOne_formatting_sample_title_authors_affiliations.pdf](https://track.editorialmanager.com/CL0/https:%2F%2Fjournals.plos.org%2Fplosone%2Fs%2Ffile%3Fid=ba62%2FPLOSOne_formatting_sample_title_authors_affiliations.pdf/1/010f019d6117a4df-f456d06a-45cc-4755-b303-5bc0431b0a28-000000/VEIyCrhpec7IL8Or9A9AcinPHmj7UaaIbMovaRf09JY=257)

*We have done our best to meet the style requirements detailed in the above links. We hope this is satisfactory.*

2. Please provide a complete Data Availability Statement in the submission form, ensuring you include all necessary access information or a reason for why you are unable to make your data freely accessible. If your research concerns only data provided within your submission, please write "All data are in the manuscript and/or supporting information files" as your Data Availability Statement.

*We have included additional information in our Data Availability Statement in the manuscript and the submission form. We hope this will suffice.*

3. We note that you have indicated that there are restrictions to data sharing for this study. For studies involving human research participant data or other sensitive data, we encourage authors to share de-identified or anonymized data. However, when data cannot be publicly shared for ethical reasons, we allow authors to make their data sets available upon request. For information on unacceptable data access restrictions, please see http://journals.plos.org/plosone/s/data-availability#loc-unacceptable-data-access-restrictions.

Before we proceed with your manuscript, please address the following prompts:

a) If there are ethical or legal restrictions on sharing a de-identified data set, please explain them in detail (e.g., data contain potentially identifying or sensitive patient information, data are owned by a third-party organization, etc.) and who has imposed them (e.g., a Research Ethics Committee or Institutional Review Board, etc.). Please also provide contact information for a data access committee, ethics committee, or other institutional body to which data requests may be sent.

*We have done our best to abide by this requirement. We hope this is sufficiently done.*

b) If there are no restrictions, please upload the minimal anonymized data set necessary to replicate your study findings to a stable, public repository and provide us with the relevant URLs, DOIs, or accession numbers. Please see http://www.bmj.com/content/340/bmj.c181.long for guidelines on how to de-identify and prepare clinical data for publication. For a list of recommended repositories, please see https://journals.plos.org/plosone/s/recommended-repositories. You also have the option of uploading the data as Supporting Information files, but we would recommend depositing data directly to a data repository if possible.

Please update your Data Availability statement in the submission form accordingly.

4. Thank you for stating the following in the Competing Interests section:
VK has received coverage for congress travel/accommodation expenses from Pfizer. JO has been a scientific advisor (advisory committee) to Astra-Zeneca, GlaxoSmithKline, MSD Finland, and Pfizer; received lecture honoraria from Advanz Pharma, Biocodex, GlaxoSmithKline, Pfizer, Professio, Roche, and Tillotts; and received coverage for congress travel/accommodation expenses from Gilead and Unimedic Pharma.

Please confirm that this does not alter your adherence to all PLOS ONE policies on sharing data and materials, by including the following statement: "This does not alter our adherence to PLOS ONE policies on sharing data and materials.” (as detailed online in our guide for authors http://journals.plos.org/plosone/s/competing-interests). If there are restrictions on sharing of data and/or materials, please state these. Please note that we cannot proceed with consideration of your article until this information has been declared.

*We have updated the Competing Interests section of the manuscript, as well as the new cover letter.*

Please include your updated Competing Interests statement in your cover letter; we will change the online submission form on your behalf.

5. If the reviewer comments include a recommendation to cite specific previously published works, please review and evaluate these publications to determine whether they are relevant and should be cited. There is no requirement to cite these works unless the editor has indicated otherwise. 


[Note: HTML markup is below. Please do not edit.]

Reviewers' comments:

Reviewer's Responses to Questions

**Comments to the Author**

1. Is the manuscript technically sound, and do the data support the conclusions?

The manuscript must describe a technically sound piece of scientific research with data that supports the conclusions. Experiments must have been conducted rigorously, with appropriate controls, replication, and sample sizes. The conclusions must be drawn appropriately based on the data presented.

Reviewer #1: Partly

Reviewer #2: Yes

2. Has the statistical analysis been performed appropriately and rigorously?

Reviewer #1: No

Reviewer #2: Yes

3. Have the authors made all data underlying the findings in their manuscript fully available?

The [PLOS Data policy](http://track.editorialmanager.com/CL0/http:%2F%2Fwww.plosone.org%2Fstatic%2Fpolicies.action%23sharing/1/010f019d6117a4df-f456d06a-45cc-4755-b303-5bc0431b0a28-000000/G21IZw1dNe1A1YrPWz0KVBL_h5v-0avoqNtEnCAJWEs=257) requires authors to make all data underlying the findings described in their manuscript fully available without restriction, with rare exception (please refer to the Data Availability Statement in the manuscript PDF file). The data should be provided as part of the manuscript or its supporting information, or deposited to a public repository. For example, in addition to summary statistics, the data points behind means, medians and variance measures should be available. If there are restrictions on publicly sharing data—e.g. participant privacy or use of data from a third party—those must be specified.

Reviewer #1: Yes

Reviewer #2: Yes

4. Is the manuscript presented in an intelligible fashion and written in standard English?

PLOS ONE does not copyedit accepted manuscripts, so the language in submitted articles must be clear, correct, and unambiguous. Any typographical or grammatical errors should be corrected at revision, so please note any specific errors here.

Reviewer #1: Yes

Reviewer #2: Yes

5. Review Comments to the Author

Please use the space provided to explain your answers to the questions above. You may also include additional comments for the author, including concerns about dual publication, research ethics, or publication ethics. (Please upload your review as an attachment if it exceeds 20,000 characters)

Reviewer #1: Comments
• Only four patients experienced delayed death (after timepoint B), yet multiple gene modules, individual genes, and HLA types are evaluated in relation to this outcome. The authors themselves cite Peduzzi et al. and acknowledge that the events-per-variable ratio required for robust modeling is not met. In its current form, the risk of overfitting and chance associations is very high. Please substantially temper all causal and predictive claims regarding delayed mortality, explicitly frame findings as exploratory/hypothesis-generating, and avoid language implying that these modules “differentiate delayed death” with clinical reliability. The ROC/ AUC values of 0.95–1.00 for individual genes/modules with only four events are not credible as stable estimates. These require clear reporting of CI, emphasis on overfitting and optimism bias and removal of any suggestion that these are ready for clinical risk stratification.

*We thank the reviewer for this important comment and apologize for sounding too enthused about our findings. We have attempted to adjust the language of the manuscript to better reflect the uncertainties pertaining to small sample size and lack of predictive power. All mentions of differentiation as compared to CRP values have been removed, and the Discussion has been duly edited at lines 215-217, 223-229, 236-238, 253-254. Also the Conclusions section has been amended with wording that hopefully is more tempered, at lines 262-265. We hope this is sufficient.*

• Patients who died after timepoint B were significantly older and had markedly higher Charlson comorbidity scores than survivors (mean age 78 vs 53 years, Charlson 7.5 vs 2.4). These factors are strong predictors of delayed mortality and may drive both the clinical outcome and aspects of the transcriptome. The current analysis appears largely unadjusted for these key confounders.

*We appreciate the reviewer raising this valid point. We have attempted to accommodate this by editing Figure 1 to include categories for age and Charlson comorbidity index. The legend has been amended to describe this at lines 164-166. We have also attempted to further elaborate this point in the Discussion section at lines 243-248. We hope this is satisfactory.*

• The Abstract and Conclusions state that gene expression “was better at differentiating delayed death from survival than C-reactive protein level.” Yet there is no formal comparison of discrimination (e.g., AUCs with CIs for module vs CRP, or net reclassification) and CRP differences are described only descriptively and non-significant.

*We are grateful for this astute observation and are in agreement. We have thus promptly removed mentions of this differentiation, as mentioned above.*

• The Discussion notes that prior work in chronic critical illness found upregulation of many HLA genes, whereas this study finds downregulation of multiple HLA genes at timepoint B associated with severity and death. This apparent discrepancy deserves deeper exploration.

*We thank the reviewer for bringing this point up. We have attempted to discuss this matter further in the Discussion section now, at lines 215-217. We hope this will be satisfactory.*

• A more nuanced, mechanistic discussion would improve the translational value and help avoid overinterpretation.

*We thank the reviewer for this important comment, and have attempted to improve the language and wording of our manuscript overall, as elaborated in other sections of this response.*

• The authors acknowledge that whole-blood RNA-seq cannot distinguish per-cell expression from changes in leukocyte populations. This is particularly relevant because modules linked to delayed death at timepoint B relate to neutrophil regulation, necroptosis, and HLA expression and are associated with leukocyte count and right shift.

*We appreciate the reviewer highlighting this matter and agree that it merits further discussion. We have sought to remedy this in the Discussion section at lines 248-250 and 258-259. We hope this will suffice.*

• Explore whether simple measures such as neutrophil and lymphocyte counts (or NLR) correlate with the key modules and whether modules remain associated with outcome after adjusting for these counts in a univariable or limited multivariable framework.

*We are grateful to the reviewer for this comment and have added the categories of full leukocyte count and leukocyte count minus lymphocyte count to Figure 1 to probe this matter. We have also elaborated on this, espcially regarding study limitations in the Discussion section, lines 248-250 and 258-259. We hope this is satisfactory.*

• Emphasize in the Discussion that some transcriptomic signals may primarily reflect shifts in cell populations rather than intrinsic reprogramming.

*We appreciate this important notion and have tried to accommodate it in the Discussion section. As it has to do with the previous two points, the lines are the same 248-250 and 258-259. We hope this is sufficient.*

• Provide thresholds for inclusion of genes (filtering criteria for low expression/variance) and the soft-thresholding power used in WGCNA, including rationale.

*We thank the reviewer for raising this and have added clarification at lines 96-99 and 101-103 in the Methods section. We hope this proves satisfactory.*

• Clarify module preservation/overlap between timepoints A and B; the text states that genes were reassigned at each timepoint, but some quantitative measure of overlap would help interpret the “markedly different” profiles.

*We are thankful for this important comment and have attempted to make the necessary additions to the Results section at line 154 and lines 180-185, and also added lines 223-224 to the Discussion section, as well as softened the wording of the Conclusion to reflect this matter. We hope this will be suffcient.*

• Multiple testing: clarify how multiple comparisons were controlled across modules, traits, and genes (beyond the -log10(P)*sign(r) metric). Were adjusted p-values or FDRs calculated for module–trait associations and GO analyses?

*We thank the reviewer for this comment, and have clarified in the legend at line 161 that the p-values are unadjusted. We used the unadjusted value, as historically a non-adjusted p-value was used, e.g., also in the original tutorial of the WGCNA developers, and we have followed their instructions. We hope this is satisfactory.*

• For the Mann–Whitney U tests comparing laboratory values between survivors and delayed deaths, provide exact p-values and 95% CIs where possible.

*We appreciate this important comment and have added the requested values at lines 135-140.*

• Please clarify:
• The distribution of time from symptom onset and from admission to sampling at timepoints A and B (medians and IQRs).

*We are grateful tor this sound point and have added this information at lines 124-127.*

• How deaths occurring “later in hospital” and “over a month after onset” are allocated into early vs delayed categories.

*We thank the reviewer for this pertinent comment. All deaths occurring after timepoint B were in the delayed death category, while all deaths before timepoint B were early deaths. We have attempted to clarify this in the Definitions section at line 118 and in the Results section, at lines 132-133. We hope this is satisfactory.*

• Whether any patients were lost to follow-up between discharge and 90 days, and how this was handled.

*We appreciate this important consideration and have clarified this in the Results section at lines 133-134.*

• For Table 1, report 95% CIs for the AUCs and sample sizes used.

*We are grateful for this pertinent comment. We have added the requested confidence intervals to Table 1, and the sample size (the four cases who perished after timepoint B) to the legend at lines 189-190. We hope this is sufficient.*

• For Table 2, briefly indicate how GO enrichment p-values were adjusted (e.g., Benjamini–Hochberg).

*We thank the reviewer for this comment. The adjusting was indeed done with Benjamini-Hochberg. This has been clarified in the Methods section at lines 101-103 as well as the Table legend at line 196. We hope this will satisfy.*

Recommendation
In summary, the study raises an interesting and clinically relevant hypothesis about distinct transcriptomic patterns associated with delayed mortality in iGAS sepsis, but the very limited number of delayed death events and lack of robust adjustment for confounding restrict the strength of the conclusions. I would support publication in PLOS ONE after major revision that (1) reframes the work as exploratory, (2) clearly presents the limitations and potential biases, and (3) strengthens methodological transparency and interpretative restraint as outlined above.

*We again thank the reviewer for a most insightful and thorough commentary on our paper. We have tried to accommodate the requests, as detailed above, and hope this proves satisfactory. We feel this has greatly improved our paper.*

Reviewer #2: This is an interesting exploratory study of possible associations between gene expression as determined by transcriptomics in peripheral blood and outcome, specifically delayed mortality, in patients with iGAS. The sample collection was made as part of a prospective observational study of iGAS, which is a commendable effort. 45 included patients is a good sample size considering the relative rarity of iGAS, at least for a nordic setting, although it restricts the possibilities for multivariable analysis. The deceased patients were, as expected, significantly older and with more co-mordbidity. There were also indications of an increased and sustained systemic inflammatory response in patients with poor outcome. The results provide clues to the mechanisms linking advanced age and co-morbidity to worse prognosis through a dysregulated immune response. However, iGAS is an heterogenous disease and there are more potential factors that may also come into play, such as bacterial load, type of infection focus, patient's delay etc. The authors acknowledge this in their discussion. The results are presented clearly and conclusions are supported by the provided data.

Minor comments
1. I think the term "right shift" is somewhat ambiguous. Presumably, the authors mean low lymphocyte count relative to total leukocyte count. In my experience, right shift can also be related to the distribution of mature vs immature neutrophiles. I would advise to use a more precise term

*We thank the reviewer for this clarification, and have amended all mentions of “right shift” to “total leukocyte count minus lymphocyte count”, which was what was actually measured. We hope this will be satisfactory.*

2. The time point of sampling is stated as 2 days after admission and 5-7 days later. There is no data on time point in relation to first day of symptoms, although disease duration could potentially influence results and interpretation.

*We appreciate this important comment and have added clarification on this in the Results section at lines 124-127, as well as lines 142-143. We hope this is sufficient.*

3. Were there any associations between infection focus and gene expression?

*We thank the reviewer for this question. We did check this, but as the case numbers for separate infection foci were small, no significant associations arose. This information has been added to the Results section, at line 156.*
