# Supplementary material for: Transcriptome profile of delayed mortality in patients with invasive group A Streptococcal disease
Source: PLoS One. 2026 Aug 18;21(8):e0356286. doi: 10.1371/journal.pone.0356286 (PMC13484954; doi:10.1371/journal.pone.0356286)
Supplement: Supplementary file 3 [file pone.0356286.s004.docx]

**Response to reviewer**

*Dear editor*

*Below you will find our response to our reviewers comments. They are written in italics to stand out.*

Remaining concerns

 Despite the revisions, the number of delayed deaths remains very small, so any predictive interpretation should remain strictly exploratory.

 The reported discrimination statistics still need to be interpreted with caution because small-event ROC estimates can be unstable and optimistic.

 The manuscript would benefit from even clearer acknowledgment that associations are not evidence of causality and should not be presented as clinically actionable biomarkers at this stage.

*We are grateful to the reviewer for having read our revised paper again with care, and the comments above are sound and well worder. We have done our best to accommodate them.*

*Wording has been changed in the manuscript to reflect this at lines 46, 52-55 in the Abstract, lines 217, 222, 256-258 in Discussion, and lines 266, 268-269 in Conclusions.*

 Minor points

 The replacement of “right shift” with a more precise term is appropriate and improves clarity.

 The updated description of infection foci is acceptable, and the clarification that no significant association was found is sufficient.

 The revised data-availability and competing-interests statements appear more complete and consistent with PLOS ONE requirements.

 Recommendation

 Overall, the revision is much improved and acceptable in principle, provided the final version continues to emphasize its exploratory nature and avoids overstating prediction or clinical applicability

*We thank the reviewer for this recommendation, and have tried to further emphasize the exploratory and uncertain nature of our findings by altering our wording, as mentioned above, at lines 46, 52-55 in the Abstract, lines 217, 222, 256-258 in Discussion, and lines 266, 268-269 in Conclusions.*
