# Supplementary material for: Transcriptome profile of delayed mortality in patients with invasive group A Streptococcal disease
Source: PLoS One. 2026 Aug 18;21(8):e0356286. doi: 10.1371/journal.pone.0356286 (PMC13484954; doi:10.1371/journal.pone.0356286)
Supplement: Peer review history

# Peer review: Decision Letter 0

**DOI:** 10.1371/journal.pone.0356286.r001

**Authors:** Yury Khudyakov

6 Apr 2026

Dear Dr. Kailankangas,

Thank you for submitting your manuscript to PLOS ONE. After careful consideration, we feel that it has merit but does not fully meet PLOS ONE’s publication criteria as it currently stands. Therefore, we invite you to submit a revised version of the manuscript that addresses the points raised during the review process.

==============================

Your manuscript was reviewed by two experts in the field. Both identified many important problems in your submission which require your careful attention. Please respond to all comments point-by-point.

==============================

Please submit your revised manuscript by May 21 2026 11:59PM. If you will need more time than this to complete your revisions, please reply to this message or contact the journal office at plosone@plos.org. When you're ready to submit your revision, log on to https://www.editorialmanager.com/pone/ and select the 'Submissions Needing Revision' folder to locate your manuscript file.

- A letter that responds to each point raised by the academic editor and reviewer(s). You should upload this letter as a separate file labeled 'Response to Reviewers'.
- A marked-up copy of your manuscript that highlights changes made to the original version. You should upload this as a separate file labeled 'Revised Manuscript with Track Changes'.
- An unmarked version of your revised paper without tracked changes. You should upload this as a separate file labeled 'Manuscript'.

If you would like to make changes to your financial disclosure, please include your updated statement in your cover letter. Guidelines for resubmitting your figure files are available below the reviewer comments at the end of this letter.

If applicable, we recommend that you deposit your laboratory protocols in protocols.io to enhance the reproducibility of your results. Protocols.io assigns your protocol its own identifier (DOI) so that it can be cited independently in the future. For instructions see: https://journals.plos.org/plosone/s/submission-guidelines#loc-laboratory-protocols. Additionally, PLOS ONE offers an option for publishing peer-reviewed Lab Protocol articles, which describe protocols hosted on protocols.io. Read more information on sharing protocols at https://plos.org/protocols?utm_medium=editorial-email&utm_source=authorletters&utm_campaign=protocols.

As the corresponding author, your ORCID iD is verified in the submission system and will appear in the published article. PLOS supports the use of ORCID, and we encourage all coauthors to register for an ORCID iD and use it as well. Please encourage your coauthors to verify their ORCID iD within the submission system before final acceptance, as unverified ORCID iDs will not appear in the published article. *Only*  the individual author can complete the verification step; PLOS staff *cannot*  verify ORCID iDs on behalf of authors.

We look forward to receiving your revised manuscript.

Kind regards,

Yury E Khudyakov, PhD

Academic Editor

PLOS One

Journal Requirements:

When submitting your revision, we need you to address these additional requirements.

1. Please ensure that your manuscript meets PLOS ONE's style requirements, including those for file naming. The PLOS ONE style templates can be found at

https://journals.plos.org/plosone/s/file?id=wjVg/PLOSOne_formatting_sample_main_body.pdf and https://journals.plos.org/plosone/s/file?id=ba62/PLOSOne_formatting_sample_title_authors_affiliations.pdf

2. Please provide a complete Data Availability Statement in the submission form, ensuring you include all necessary access information or a reason for why you are unable to make your data freely accessible. If your research concerns only data provided within your submission, please write "All data are in the manuscript and/or supporting information files" as your Data Availability Statement.

3. We note that you have indicated that there are restrictions to data sharing for this study. For studies involving human research participant data or other sensitive data, we encourage authors to share de-identified or anonymized data. However, when data cannot be publicly shared for ethical reasons, we allow authors to make their data sets available upon request. For information on unacceptable data access restrictions, please see http://journals.plos.org/plosone/s/data-availability#loc-unacceptable-data-access-restrictions.

Before we proceed with your manuscript, please address the following prompts:

a) If there are ethical or legal restrictions on sharing a de-identified data set, please explain them in detail (e.g., data contain potentially identifying or sensitive patient information, data are owned by a third-party organization, etc.) and who has imposed them (e.g., a Research Ethics Committee or Institutional Review Board, etc.). Please also provide contact information for a data access committee, ethics committee, or other institutional body to which data requests may be sent.

b) If there are no restrictions, please upload the minimal anonymized data set necessary to replicate your study findings to a stable, public repository and provide us with the relevant URLs, DOIs, or accession numbers. Please see http://www.bmj.com/content/340/bmj.c181.long for guidelines on how to de-identify and prepare clinical data for publication. For a list of recommended repositories, please see https://journals.plos.org/plosone/s/recommended-repositories. You also have the option of uploading the data as Supporting Information files, but we would recommend depositing data directly to a data repository if possible.

Please update your Data Availability statement in the submission form accordingly.

4. Thank you for stating the following in the Competing Interests section:

VK has received coverage for congress travel/accommodation expenses from Pfizer. JO has been a scientific advisor (advisory committee) to Astra-Zeneca, GlaxoSmithKline, MSD Finland, and Pfizer; received lecture honoraria from Advanz Pharma, Biocodex, GlaxoSmithKline, Pfizer, Professio, Roche, and Tillotts; and received coverage for congress travel/accommodation expenses from Gilead and Unimedic Pharma.

Please confirm that this does not alter your adherence to all PLOS ONE policies on sharing data and materials, by including the following statement: "This does not alter our adherence to PLOS ONE policies on sharing data and materials.” (as detailed online in our guide for authors http://journals.plos.org/plosone/s/competing-interests). If there are restrictions on sharing of data and/or materials, please state these. Please note that we cannot proceed with consideration of your article until this information has been declared.

Please include your updated Competing Interests statement in your cover letter; we will change the online submission form on your behalf.

5. If the reviewer comments include a recommendation to cite specific previously published works, please review and evaluate these publications to determine whether they are relevant and should be cited. There is no requirement to cite these works unless the editor has indicated otherwise.

[Note: HTML markup is below. Please do not edit.]

Reviewers' comments:

Reviewer's Responses to Questions

**Comments to the Author**

1. Is the manuscript technically sound, and do the data support the conclusions?

Reviewer #1: Partly

Reviewer #2: Yes

**********

2. Has the statistical analysis been performed appropriately and rigorously?

Reviewer #1: No

Reviewer #2: Yes

**********

3. Have the authors made all data underlying the findings in their manuscript fully available?

The PLOS Data policy

Reviewer #1: Yes

Reviewer #2: Yes

**********

4. Is the manuscript presented in an intelligible fashion and written in standard English?

Reviewer #1: Yes

Reviewer #2: Yes

**********

Reviewer #1: Comments

• Only four patients experienced delayed death (after timepoint B), yet multiple gene modules, individual genes, and HLA types are evaluated in relation to this outcome. The authors themselves cite Peduzzi et al. and acknowledge that the events-per-variable ratio required for robust modeling is not met. In its current form, the risk of overfitting and chance associations is very high. Please substantially temper all causal and predictive claims regarding delayed mortality, explicitly frame findings as exploratory/hypothesis-generating, and avoid language implying that these modules “differentiate delayed death” with clinical reliability. The ROC/ AUC values of 0.95–1.00 for individual genes/modules with only four events are not credible as stable estimates. These require clear reporting of CI, emphasis on overfitting and optimism bias and removal of any suggestion that these are ready for clinical risk stratification.

• Patients who died after timepoint B were significantly older and had markedly higher Charlson comorbidity scores than survivors (mean age 78 vs 53 years, Charlson 7.5 vs 2.4). These factors are strong predictors of delayed mortality and may drive both the clinical outcome and aspects of the transcriptome. The current analysis appears largely unadjusted for these key confounders.

• The Abstract and Conclusions state that gene expression “was better at differentiating delayed death from survival than C-reactive protein level.” Yet there is no formal comparison of discrimination (e.g., AUCs with CIs for module vs CRP, or net reclassification) and CRP differences are described only descriptively and non-significant.

• The Discussion notes that prior work in chronic critical illness found upregulation of many HLA genes, whereas this study finds downregulation of multiple HLA genes at timepoint B associated with severity and death. This apparent discrepancy deserves deeper exploration.

• A more nuanced, mechanistic discussion would improve the translational value and help avoid overinterpretation.

• The authors acknowledge that whole-blood RNA-seq cannot distinguish per-cell expression from changes in leukocyte populations. This is particularly relevant because modules linked to delayed death at timepoint B relate to neutrophil regulation, necroptosis, and HLA expression and are associated with leukocyte count and right shift.

• Explore whether simple measures such as neutrophil and lymphocyte counts (or NLR) correlate with the key modules and whether modules remain associated with outcome after adjusting for these counts in a univariable or limited multivariable framework.

• Emphasize in the Discussion that some transcriptomic signals may primarily reflect shifts in cell populations rather than intrinsic reprogramming.

• Provide thresholds for inclusion of genes (filtering criteria for low expression/variance) and the soft-thresholding power used in WGCNA, including rationale.

• Clarify module preservation/overlap between timepoints A and B; the text states that genes were reassigned at each timepoint, but some quantitative measure of overlap would help interpret the “markedly different” profiles.

• Multiple testing: clarify how multiple comparisons were controlled across modules, traits, and genes (beyond the -log10(P)*sign(r) metric). Were adjusted p-values or FDRs calculated for module–trait associations and GO analyses?

• For the Mann–Whitney U tests comparing laboratory values between survivors and delayed deaths, provide exact p-values and 95% CIs where possible.

• Please clarify:

• The distribution of time from symptom onset and from admission to sampling at timepoints A and B (medians and IQRs).

• How deaths occurring “later in hospital” and “over a month after onset” are allocated into early vs delayed categories.

• Whether any patients were lost to follow-up between discharge and 90 days, and how this was handled.

• For Table 1, report 95% CIs for the AUCs and sample sizes used.

• For Table 2, briefly indicate how GO enrichment p-values were adjusted (e.g., Benjamini–Hochberg).

Recommendation

In summary, the study raises an interesting and clinically relevant hypothesis about distinct transcriptomic patterns associated with delayed mortality in iGAS sepsis, but the very limited number of delayed death events and lack of robust adjustment for confounding restrict the strength of the conclusions. I would support publication in PLOS ONE after major revision that (1) reframes the work as exploratory, (2) clearly presents the limitations and potential biases, and (3) strengthens methodological transparency and interpretative restraint as outlined above.

Reviewer #2: This is an interesting exploratory study of possible associations between gene expression as determined by transcriptomics in peripheral blood and outcome, specifically delayed mortality, in patients with iGAS. The sample collection was made as part of a prospective observational study of iGAS, which is a commendable effort. 45 included patients is a good sample size considering the relative rarity of iGAS, at least for a nordic setting, although it restricts the possibilities for multivariable analysis. The deceased patients were, as expected, significantly older and with more co-mordbidity. There were also indications of an increased and sustained systemic inflammatory response in patients with poor outcome. The results provide clues to the mechanisms linking advanced age and co-morbidity to worse prognosis through a dysregulated immune response. However, iGAS is an heterogenous disease and there are more potential factors that may also come into play, such as bacterial load, type of infection focus, patient's delay etc. The authors acknowledge this in their discussion. The results are presented clearly and conclusions are supported by the provided data.

Minor comments

1. I think the term "right shift" is somewhat ambiguous. Presumably, the authors mean low lymphocyte count relative to total leukocyte count. In my experience, right shift can also be related to the distribution of mature vs immature neutrophiles. I would advise to use a more precise term

2. The time point of sampling is stated as 2 days after admission and 5-7 days later. There is no data on time point in relation to first day of symptoms, although disease duration could potentially influence results and interpretation.

3. Were there any associations between infection focus and gene expression?

**********

what does this mean?). If published, this will include your full peer review and any attached files.

If you choose “no”, your identity will remain anonymous but your review may still be made public.

**Do you want your identity to be public for this peer review?**  For information about this choice, including consent withdrawal, please see our Privacy Policy

Reviewer #1: **Yes:**  Atul Jindal

Reviewer #2: No

**********

[NOTE: If reviewer comments were submitted as an attachment file, they will be attached to this email and accessible via the submission site. Please log into your account, locate the manuscript record, and check for the action link "View Attachments". If this link does not appear, there are no attachment files.]

To ensure your figures meet our technical requirements, please review our figure guidelines: https://journals.plos.org/plosone/s/figures

You may also use PLOS’s free figure tool, NAAS, to help you prepare publication quality figures: https://journals.plos.org/plosone/s/figures#loc-tools-for-figure-preparation.

NAAS will assess whether your figures meet our technical requirements by comparing each figure against our figure specifications.

Attachment Submitted filename: Plos One Comments.docx

# Author comment: Author response to Decision Letter 1

**DOI:** 10.1371/journal.pone.0356286.r002

20 Jun 2026

1. Please ensure that your manuscript meets PLOS ONE's style requirements, including those for file naming. The PLOS ONE style templates can be found at

https://journals.plos.org/plosone/s/file?id=wjVg/PLOSOne_formatting_sample_main_body.pdf and https://journals.plos.org/plosone/s/file?id=ba62/PLOSOne_formatting_sample_title_authors_affiliations.pdf

We have done our best to meet the style requirements detailed in the above links. We hope this is satisfactory.

2. Please provide a complete Data Availability Statement in the submission form, ensuring you include all necessary access information or a reason for why you are unable to make your data freely accessible. If your research concerns only data provided within your submission, please write "All data are in the manuscript and/or supporting information files" as your Data Availability Statement.

We have included additional information in our Data Availability Statement in the manuscript and the submission form. We hope this will suffice.

3. We note that you have indicated that there are restrictions to data sharing for this study. For studies involving human research participant data or other sensitive data, we encourage authors to share de-identified or anonymized data. However, when data cannot be publicly shared for ethical reasons, we allow authors to make their data sets available upon request. For information on unacceptable data access restrictions, please see http://journals.plos.org/plosone/s/data-availability#loc-unacceptable-data-access-restrictions.

Before we proceed with your manuscript, please address the following prompts:

a) If there are ethical or legal restrictions on sharing a de-identified data set, please explain them in detail (e.g., data contain potentially identifying or sensitive patient information, data are owned by a third-party organization, etc.) and who has imposed them (e.g., a Research Ethics Committee or Institutional Review Board, etc.). Please also provide contact information for a data access committee, ethics committee, or other institutional body to which data requests may be sent.

We have done our best to abide by this requirement. We hope this is sufficiently done.

b) If there are no restrictions, please upload the minimal anonymized data set necessary to replicate your study findings to a stable, public repository and provide us with the relevant URLs, DOIs, or accession numbers. Please see http://www.bmj.com/content/340/bmj.c181.long for guidelines on how to de-identify and prepare clinical data for publication. For a list of recommended repositories, please see https://journals.plos.org/plosone/s/recommended-repositories. You also have the option of uploading the data as Supporting Information files, but we would recommend depositing data directly to a data repository if possible.

Please update your Data Availability statement in the submission form accordingly.

4. Thank you for stating the following in the Competing Interests section:

VK has received coverage for congress travel/accommodation expenses from Pfizer. JO has been a scientific advisor (advisory committee) to Astra-Zeneca, GlaxoSmithKline, MSD Finland, and Pfizer; received lecture honoraria from Advanz Pharma, Biocodex, GlaxoSmithKline, Pfizer, Professio, Roche, and Tillotts; and received coverage for congress travel/accommodation expenses from Gilead and Unimedic Pharma.

Please confirm that this does not alter your adherence to all PLOS ONE policies on sharing data and materials, by including the following statement: "This does not alter our adherence to PLOS ONE policies on sharing data and materials.” (as detailed online in our guide for authors http://journals.plos.org/plosone/s/competing-interests). If there are restrictions on sharing of data and/or materials, please state these. Please note that we cannot proceed with consideration of your article until this information has been declared.

We have updated the Competing Interests section of the manuscript, as well as the new cover letter.

Please include your updated Competing Interests statement in your cover letter; we will change the online submission form on your behalf.

5. If the reviewer comments include a recommendation to cite specific previously published works, please review and evaluate these publications to determine whether they are relevant and should be cited. There is no requirement to cite these works unless the editor has indicated otherwise.

[Note: HTML markup is below. Please do not edit.]

Reviewers' comments:

Reviewer's Responses to Questions

Comments to the Author

1. Is the manuscript technically sound, and do the data support the conclusions?

The manuscript must describe a technically sound piece of scientific research with data that supports the conclusions. Experiments must have been conducted rigorously, with appropriate controls, replication, and sample sizes. The conclusions must be drawn appropriately based on the data presented.

Reviewer #1: Partly

Reviewer #2: Yes

Muoto

2. Has the statistical analysis been performed appropriately and rigorously?

Reviewer #1: No

Reviewer #2: Yes

Muoto

3. Have the authors made all data underlying the findings in their manuscript fully available?

The PLOS Data policy requires authors to make all data underlying the findings described in their manuscript fully available without restriction, with rare exception (please refer to the Data Availability Statement in the manuscript PDF file). The data should be provided as part of the manuscript or its supporting information, or deposited to a public repository. For example, in addition to summary statistics, the data points behind means, medians and variance measures should be available. If there are restrictions on publicly sharing data—e.g. participant privacy or use of data from a third party—those must be specified.

Reviewer #1: Yes

Reviewer #2: Yes

Muoto

4. Is the manuscript presented in an intelligible fashion and written in standard English?

PLOS ONE does not copyedit accepted manuscripts, so the language in submitted articles must be clear, correct, and unambiguous. Any typographical or grammatical errors should be corrected at revision, so please note any specific errors here.

Reviewer #1: Yes

Reviewer #2: Yes

Muoto

5. Review Comments to the Author

Please use the space provided to explain your answers to the questions above. You may also include additional comments for the author, including concerns about dual publication, research ethics, or publication ethics. (Please upload your review as an attachment if it exceeds 20,000 characters)

Reviewer #1: Comments

• Only four patients experienced delayed death (after timepoint B), yet multiple gene modules, individual genes, and HLA types are evaluated in relation to this outcome. The authors themselves cite Peduzzi et al. and acknowledge that the events-per-variable ratio required for robust modeling is not met. In its current form, the risk of overfitting and chance associations is very high. Please substantially temper all causal and predictive claims regarding delayed mortality, explicitly frame findings as exploratory/hypothesis-generating, and avoid language implying that these modules “differentiate delayed death” with clinical reliability. The ROC/ AUC values of 0.95–1.00 for individual genes/modules with only four events are not credible as stable estimates. These require clear reporting of CI, emphasis on overfitting and optimism bias and removal of any suggestion that these are ready for clinical risk stratification.

We thank the reviewer for this important comment and apologize for sounding too enthused about our findings. We have attempted to adjust the language of the manuscript to better reflect the uncertainties pertaining to small sample size and lack of predictive power. All mentions of differentiation as compared to CRP values have been removed, and the Discussion has been duly edited at lines 215-217, 223-229, 236-238, 253-254. Also the Conclusions section has been amended with wording that hopefully is more tempered, at lines 262-265. We hope this is sufficient.

• Patients who died after timepoint B were significantly older and had markedly higher Charlson comorbidity scores than survivors (mean age 78 vs 53 years, Charlson 7.5 vs 2.4). These factors are strong predictors of delayed mortality and may drive both the clinical outcome and aspects of the transcriptome. The current analysis appears largely unadjusted for these key confounders.

We appreciate the reviewer raising this valid point. We have attempted to accommodate this by editing Figure 1 to include categories for age and Charlson comorbidity index. The legend has been amended to describe this at lines 164-166. We have also attempted to further elaborate this point in the Discussion section at lines 243-248. We hope this is satisfactory.

• The Abstract and Conclusions state that gene expression “was better at differentiating delayed death from survival than C-reactive protein level.” Yet there is no formal comparison of discrimination (e.g., AUCs with CIs for module vs CRP, or net reclassification) and CRP differences are described only descriptively and non-significant.

We are grateful for this astute observation and are in agreement. We have thus promptly removed mentions of this differentiation, as mentioned above.

• The Discussion notes that prior work in chronic critical illness found upregulation of many HLA genes, whereas this study finds downregulation of multiple HLA genes at timepoint B associated with severity and death. This apparent discrepancy deserves deeper exploration.

We thank the reviewer for bringing this point up. We have attempted to discuss this matter further in the Discussion section now, at lines 215-217. We hope this will be satisfactory.

• A more nuanced, mechanistic discussion would improve the translational value and help avoid overinterpretation.

We thank the reviewer for this important comment, and have attempted to improve the language and wording of our manuscript overall, as elaborated in other sections of this response.

• The authors acknowledge that whole-blood RNA-seq cannot distinguish per-cell expression from changes in leukocyte populations. This is particularly relevant because modules linked to delayed death at timepoint B relate to neutrophil regulation, necroptosis, and HLA expression and are associated with leukocyte count and right shift.

We appreciate the reviewer highlighting this matter and agree that it merits further discussion. We have sought to remedy this in the Discussion section at lines 248-250 and 258-259. We hope this will suffice.

• Explore whether simple measures such as neutrophil and lymphocyte counts (or NLR) correlate with the key modules and whether modules remain associated with outcome after adjusting for these counts in a univariable or limited multivariable framework.

We are grateful to the reviewer for this comment and have added the categories of full leukocyte count and leukocyte count minus lymphocyte count to Figure 1 to probe this matter. We have also elaborated on this, espcially regarding study limitations in the Discussion section, lines 248-250 and 258-259. We hope this is satisfactory.

• Emphasize in the Discussion that some transcriptomic signals may primarily reflect shifts in cell populations rather than intrinsic reprogramming.

We appreciate this important notion and have tried to accommodate it in the Discussion section. As it has to do with the previous two points, the lines are the same 248-250 and 258-259. We hope this is sufficient.

• Provide thresholds for inclusion of genes (filtering criteria for low expression/variance) and the soft-thresholding power used in WGCNA, including rationale.

We thank the reviewer for raising this and have added clarification at lines 96-99 and 101-103 in the Methods section. We hope this proves satisfactory.

• Clarify module preservation/overlap between timepoints A and B; the text states that genes were reassigned at each timepoint, but some quantitative measure of overlap would help interpret the “markedly different” profiles.

We are thankful for this important comment and have attempted to make the necessary additions to the Results section at line 154 and lines 180-185, and also added lines 223-224 to the Discussion section, as well as softened the wording of the Conclusion to reflect this matter. We hope this will be suffcient.

• Multiple testing: clarify how multiple comparisons were controlled across modules, traits, and genes (beyond the -log10(P)*sign(r) metric). Were adjusted p-values or FDRs calculated for module–trait associations and GO analyses?

We thank the reviewer for this comment, and have clarified in the legend at line 161 that the p-values are unadjusted. We used the unadjusted value, as historically a non-adjusted p-value was used, e.g., also in the original tutorial of the WGCNA developers, and we have followed their instructions. We hope this is satisfactory.

• For the Mann–Whitney U tests comparing laboratory values between survivors and delayed deaths, provide exact p-values and 95% CIs where possible.

We appreciate this important comment and have added the requested values at lines 135-140.

• Please clarify:

• The distribution of time from symptom onset and from admission to sampling at timepoints A and B (medians and IQRs).

We are grateful tor this sound point and have added this information at lines 124-127.

• How deaths occurring “later in hospital” and “over a month after onset” are allocated into early vs delayed categories.

We thank the reviewer for this pertinent comment. All deaths occurring after timepoint B were in the delayed death category, while all deaths before timepoint B were early deaths. We have attempted to clarify this in the Definitions section at line 118 and in the Results section, at lines 132-133. We hope this is satisfactory.

• Whether any patients were lost to follow-up between discharge and 90 days, and how this was handled.

We appreciate this important consideration and have clarified this in the Results section at lines 133-134.

• For Table 1, report 95% CIs for the AUCs and sample sizes used.

We are grateful for this pertinent comment. We have added the requested confidence intervals to Table 1, and the sample size (the four cases who perished after timepoint B) to the legend at lines 189-190. We hope this is sufficient.

• For Table 2, briefly indicate how GO enrichment p-values were adjusted (e.g., Benjamini–Hochberg).

We thank the reviewer for this comment. The adjusting was indeed done with Benjamini-Hochberg. This has been clarified in the Methods section at lines 101-103 as well as the Table legend at line 196. We hope this will satisfy.

Recommendation

In summary, the study raises an interesting and clinically relevant hypothesis about distinct transcriptomic patterns associated with delayed mortality in iGAS sepsis, but the very limited number of delayed death events and lack of robust adjustment for confounding restrict the strength of the conclusions. I would support publication in PLOS ONE after major revision that (1) reframes the work as exploratory, (2) clearly presents the limitations and potential biases, and (3) strengthens methodological transparency and interpretative restraint as outlined above.

We again thank the reviewer for a most insightful and thorough commentary on our paper. We have tried to accommodate the requests, as detailed above, and hope this proves satisfactory. We feel this has greatly improved our paper.

Reviewer #2: This is an interesting exploratory study of possible associations between gene expression as determined by transcriptomics in peripheral blood and outcome, specifically delayed mortality, in patients with iGAS. The sample collection was made as part of a prospective observational study of iGAS, which is a commendable effort. 45 included patients is a good sample size considering the relative r

Attachment Submitted filename: Responses to reviewers PLOS.docx

# Peer review: Decision Letter 1

**DOI:** 10.1371/journal.pone.0356286.r003

**Authors:** Yury Khudyakov

15 Jul 2026

Dear Dr. Kailankangas,

Thank you for submitting your manuscript to PLOS ONE. After careful consideration, we feel that it has merit but does not fully meet PLOS ONE’s publication criteria as it currently stands. Therefore, we invite you to submit a revised version of the manuscript that addresses the points raised during the review process.

==============================

Your revised manuscript was reviewed by one original reviewer who identified some remaining issues in your submission. Please consider the attached comments and provide point-by-point responses.

==============================

Please submit your revised manuscript by Aug 29 2026 11:59PM. If you will need more time than this to complete your revisions, please reply to this message or contact the journal office at plosone@plos.org. When you're ready to submit your revision, log on to https://www.editorialmanager.com/pone/ and select the 'Submissions Needing Revision' folder to locate your manuscript file.

- A letter that responds to each point raised by the academic editor and reviewer(s). You should upload this letter as a separate file labeled 'Response to Reviewers'.
- A marked-up copy of your manuscript that highlights changes made to the original version. You should upload this as a separate file labeled 'Revised Manuscript with Track Changes'.
- An unmarked version of your revised paper without tracked changes. You should upload this as a separate file labeled 'Manuscript'.

If you would like to make changes to your financial disclosure, please include your updated statement in your cover letter. Guidelines for resubmitting your figure files are available below the reviewer comments at the end of this letter.

If applicable, we recommend that you deposit your laboratory protocols in protocols.io to enhance the reproducibility of your results. Protocols.io assigns your protocol its own identifier (DOI) so that it can be cited independently in the future. For instructions see: https://journals.plos.org/plosone/s/submission-guidelines#loc-laboratory-protocols. Additionally, PLOS ONE offers an option for publishing peer-reviewed Lab Protocol articles, which describe protocols hosted on protocols.io. Read more information on sharing protocols at https://plos.org/protocols?utm_medium=editorial-email&utm_source=authorletters&utm_campaign=protocols.

As the corresponding author, your ORCID iD is verified in the submission system and will appear in the published article. PLOS supports the use of ORCID, and we encourage all coauthors to register for an ORCID iD and use it as well. Please encourage your coauthors to verify their ORCID iD within the submission system before final acceptance, as unverified ORCID iDs will not appear in the published article. *Only*  the individual author can complete the verification step; PLOS staff *cannot*  verify ORCID iDs on behalf of authors.

We look forward to receiving your revised manuscript.

Kind regards,

Yury E Khudyakov, PhD

Academic Editor

PLOS One

Journal Requirements:

If the reviewer comments include a recommendation to cite specific previously published works, please review and evaluate these publications to determine whether they are relevant and should be cited. There is no requirement to cite these works unless the editor has indicated otherwise.

Please review your reference list to ensure that it is complete and correct. If you have cited papers that have been retracted, please include the rationale for doing so in the manuscript text, or remove these references and replace them with relevant current references. Any changes to the reference list should be mentioned in the rebuttal letter that accompanies your revised manuscript. If you need to cite a retracted article, indicate the article’s retracted status in the References list and also include a citation and full reference for the retraction notice.

[Note: HTML markup is below. Please do not edit.]

Reviewers' comments:

Reviewer's Responses to Questions

**Comments to the Author**

Reviewer #1: (No Response)

**********

2. Is the manuscript technically sound, and do the data support the conclusions?

Reviewer #1: (No Response)

**********

3. Has the statistical analysis been performed appropriately and rigorously?

Reviewer #1: (No Response)

**********

4. Have the authors made all data underlying the findings in their manuscript fully available?

The PLOS Data policy

Reviewer #1: Yes

**********

5. Is the manuscript presented in an intelligible fashion and written in standard English?

Reviewer #1: Yes

**********

Reviewer #1: The revised manuscript is substantially improved, and the authors have addressed many of the major methodological and interpretative concerns raised in the first round. The study remains interesting as an exploratory transcriptomic analysis of delayed mortality in iGAS, but the small number of delayed-death events continues to limit the strength of inference and the scope for clinical prediction.

Major points

The authors have appropriately softened the language throughout the manuscript and now frame the findings more cautiously, which is a major improvement.

The added details on WGCNA filtering, soft-thresholding, multiple-testing handling, and confidence intervals improve methodological transparency.

The clarification of the timing of sampling relative to symptom onset and the distinction between early and delayed deaths is helpful and should reduce ambiguity.

The discussion of confounding by age, comorbidity, and leukocyte composition is more balanced, but the manuscript still cannot fully disentangle intrinsic transcriptomic reprogramming from changes in circulating cell populations.

Remaining concerns

Despite the revisions, the number of delayed deaths remains very small, so any predictive interpretation should remain strictly exploratory.

The reported discrimination statistics still need to be interpreted with caution because small-event ROC estimates can be unstable and optimistic.

The manuscript would benefit from even clearer acknowledgment that associations are not evidence of causality and should not be presented as clinically actionable biomarkers at this stage.

Minor points

The replacement of “right shift” with a more precise term is appropriate and improves clarity.

The updated description of infection foci is acceptable, and the clarification that no significant association was found is sufficient.

The revised data-availability and competing-interests statements appear more complete and consistent with PLOS ONE requirements.

Recommendation

Overall, the revision is much improved and acceptable in principle, provided the final version continues to emphasize its exploratory nature and avoids overstating prediction or clinical applicability

**********

what does this mean?). If published, this will include your full peer review and any attached files.

If you choose “no”, your identity will remain anonymous but your review may still be made public.

**Do you want your identity to be public for this peer review?**  For information about this choice, including consent withdrawal, please see our Privacy Policy

Reviewer #1: **Yes:**  Atul Jindal

**********

[NOTE: If reviewer comments were submitted as an attachment file, they will be attached to this email and accessible via the submission site. Please log into your account, locate the manuscript record, and check for the action link "View Attachments". If this link does not appear, there are no attachment files.]

To ensure your figures meet our technical requirements, please review our figure guidelines: https://journals.plos.org/plosone/s/figures

You may also use PLOS’s free figure tool, NAAS, to help you prepare publication quality figures: https://journals.plos.org/plosone/s/figures#loc-tools-for-figure-preparation.

NAAS will assess whether your figures meet our technical requirements by comparing each figure against our figure specifications.

# Author comment: Author response to Decision Letter 2

**DOI:** 10.1371/journal.pone.0356286.r004

23 Jul 2026

Remaining concerns

Despite the revisions, the number of delayed deaths remains very small, so any predictive interpretation should remain strictly exploratory.

The reported discrimination statistics still need to be interpreted with caution because small-event ROC estimates can be unstable and optimistic.

The manuscript would benefit from even clearer acknowledgment that associations are not evidence of causality and should not be presented as clinically actionable biomarkers at this stage.

We are grateful to the reviewer for having read our revised paper again with care, and the comments above are sound and well worder. We have done our best to accommodate them.

Wording has been changed in the manuscript to reflect this at lines 46, 52-55 in the Abstract, lines 217, 222, 256-258 in Discussion, and lines 266, 268-269 in Conclusions.

Minor points

The replacement of “right shift” with a more precise term is appropriate and improves clarity.

The updated description of infection foci is acceptable, and the clarification that no significant association was found is sufficient.

The revised data-availability and competing-interests statements appear more complete and consistent with PLOS ONE requirements.

Recommendation

Overall, the revision is much improved and acceptable in principle, provided the final version continues to emphasize its exploratory nature and avoids overstating prediction or clinical applicability

We thank the reviewer for this recommendation, and have tried to further emphasize the exploratory and uncertain nature of our findings by altering our wording, as mentioned above, at lines 46, 52-55 in the Abstract, lines 217, 222, 256-258 in Discussion, and lines 266, 268-269 in Conclusions.

Attachment Submitted filename: Response to reviewer.docx

# Editor report: Decision Letter 2

**DOI:** 10.1371/journal.pone.0356286.r005

**Authors:** Yury Khudyakov

2 Aug 2026

Transcriptome profile of delayed mortality in patients with invasive group A Streptococcal disease

PONE-D-25-68006R2

Dear Dr. Kailankangas,

We’re pleased to inform you that your manuscript has been judged scientifically suitable for publication and will be formally accepted for publication once it meets all outstanding technical requirements.

Within one week, you’ll receive an e-mail detailing the required amendments. When these have been addressed, you’ll receive a formal acceptance letter and your manuscript will be scheduled for publication.

An invoice will be generated when your article is formally accepted. Please note, if your institution has a publishing partnership with PLOS and your article meets the relevant criteria, all or part of your publication costs will be covered. Please make sure your user information is up-to-date by logging into Editorial Manager at Editorial Manager® and clicking the ‘Update My Information' link at the top of the page. For questions related to billing, please contact billing support.

If your institution or institutions have a press office, please notify them about your upcoming paper to help maximize its impact. If they’ll be preparing press materials, please inform our press team as soon as possible -- no later than 48 hours after receiving the formal acceptance. Your manuscript will remain under strict press embargo until 2 pm Eastern Time on the date of publication. For more information, please contact onepress@plos.org.

Kind regards,

Yury E Khudyakov, PhD

Academic Editor

PLOS One

Additional Editor Comments (optional):

Reviewers' comments:

# Editor report: Acceptance letter

**DOI:** 10.1371/journal.pone.0356286.r006

**Authors:** Yury Khudyakov

PONE-D-25-68006R2

PLOS One

Dear Dr. Kailankangas,

I'm pleased to inform you that your manuscript has been deemed suitable for publication in PLOS One. Congratulations! Your manuscript is now being handed over to our production team.

At this stage, our production department will prepare your paper for publication. This includes ensuring the following:

* All references, tables, and figures are properly cited

* All relevant supporting information is included in the manuscript submission,

* There are no issues that prevent the paper from being properly typeset

You will receive further instructions from the production team, including instructions on how to review your proof when it is ready. Please keep in mind that we are working through a large volume of accepted articles, so please give us a few days to review your paper and let you know the next and final steps.

Lastly, if your institution or institutions have a press office, please let them know about your upcoming paper now to help maximize its impact. If they'll be preparing press materials, please inform our press team within the next 48 hours. Your manuscript will remain under strict press embargo until 2 pm Eastern Time on the date of publication. For more information, please contact onepress@plos.org.

You will receive an invoice from PLOS for your publication fee after your manuscript has reached the completed accept phase. If you receive an email requesting payment before acceptance or for any other service, this may be a phishing scheme. Learn how to identify phishing emails and protect your accounts at https://explore.plos.org/phishing.

If we can help with anything else, please email us at customercare@plos.org.

Thank you for submitting your work to PLOS ONE and supporting open access.

Kind regards,

PLOS ONE Editorial Office Staff

on behalf of

Dr. Yury E Khudyakov

Academic Editor

PLOS One
